# Dataset of the analyzing trace elements and minerals via ICP-MS: Method validation for the mammalian tissue and serum samples

**DOI:** 10.1016/j.dib.2020.105218

**Published:** 2020-02-03

**Authors:** Duygu Aydemir, Gulsu Simsek, Nuriye Nuray Ulusu

**Affiliations:** a Koc University, School of Medicine, Department of Medical Biochemistry, Sariyer, 34450, Istanbul, Turkey; b Koç University Research Center for Translational Medicine (KUTTAM), Sariyer, 34450, Istanbul, Turkey; c Koç University Surface Science and Technology Center (KUYTAM), Rumelifeneri Yolu, Sariyer, 34450, Istanbul, Turkey

**Keywords:** ICP-MS, Minerals, Trace element, Mammalian tissue, Microwave digestion

## Abstract

Minerals and trace elements play vital role in the biological functions for all organisms including human and other mammals. Therefore, imbalance in the mineral and/or trace element levels may cause formation of several diseases including cancer, diabetes, cardiovascular diseases, and neurological disorders. ICP-MS (inductively coupled plasma – mass spectrometry) is described as the most sensitive and accurate method. Here we reported an effective and fast protocol as method validation to evaluate trace element and minerals via ICP-MS in the mammalian tissue and serum samples. Our data showed that minimum relative standard deviation (RSD) values with the ICP-MS were observed when we used microwave digestion with the SUPRAPUR® grade nitric acid at the lower dilution rates. Our protocol validation may help researchers to measure trace elements and minerals in the mammalian samples fast, easily and accurately. EMSURE® grade HNO_3_ caused cross contamination in the serum and tissue samples. Our protocol validation may help researchers to measure trace elements and minerals in the mammalian samples fast, easily and accurately.

**Table:** Specifications Table

Subject	Biology
Specific subject area	ICP-MS, minerals and trace elements, method validation
Type of data	Table Graph Figure
How data were acquired	ICP-MS Microwave digestion Animal experiments Statistical analysis
Data format	Raw and Analyzed
Description of data collection	Samples were digested via microwave digestion. Trace elements and minerals were measured via ICP-MS. Statistical analysis was used to compare methods.
Data source location	Medical School of Koc University, Istanbul, Turkey
Data accessibility	All data are provided in this article. Raw data is available as supplementary file.

**Table:** 

**Value of the Data** • This data compares different methods and chemicals to find most accurate technique to measure minerals and trace elements in the mammalian samples. • The data are potentially useful for researchers who need to prepare mammalian tissue and serum samples via acidic digestion for ICP-MS. • These data gave a detailed and complete set of experiments that could be used to measure Ag, Al, As, Ba, Be, Ca, Cd, Co, Cr, Cs, Cu, Fe, Ga, K, Li, Mg, Mn, Na, Ni, Pb, Rb, Se, Sr, Tl, U, V, and Zn concentrations in all types of biological samples via microwave digestion and ICP-MS.

## 1 Data

**Fig. 1:**
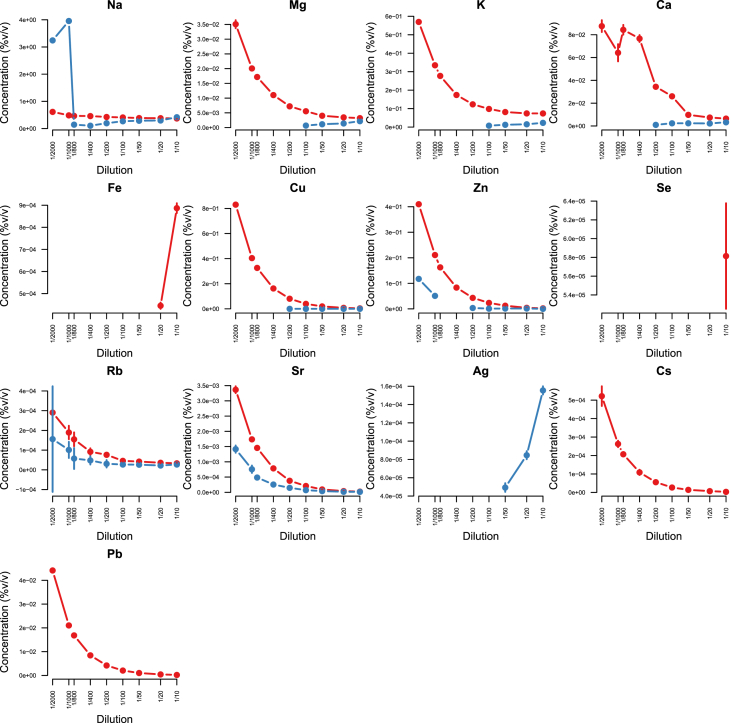
EMSURE® (red line) and SUPRAPUR® (blue line)-digested serum samples.

**Fig. 2:**
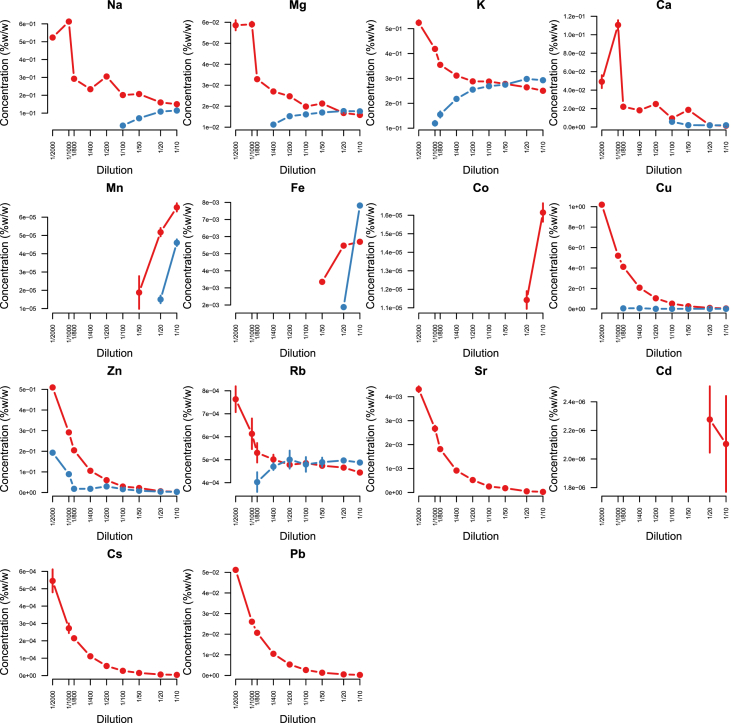
EMSURE® (red line) and SUPRAPUR® (blue line)-digested kidney samples.

**Fig. 3:**
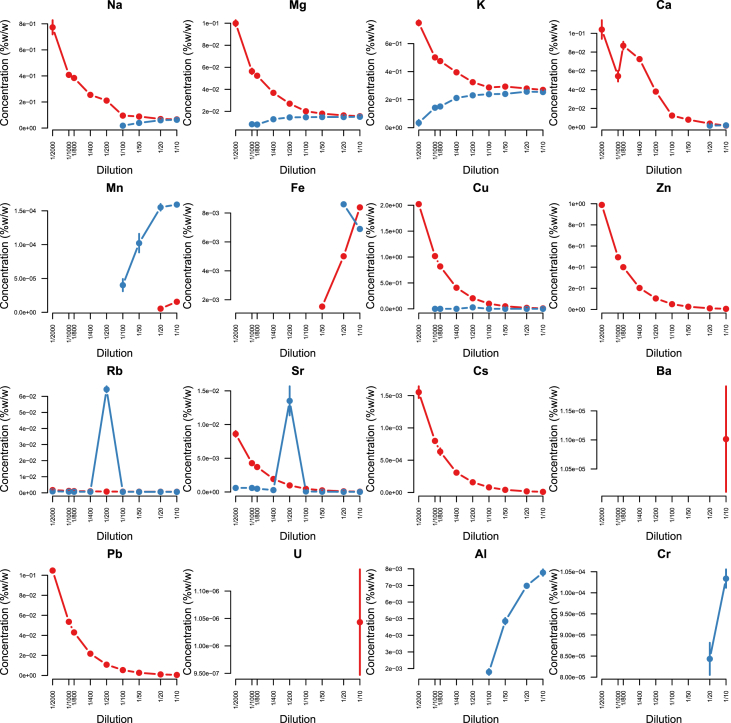
EMSURE® (red line) and SUPRAPUR® (blue line)-digested liver samples.

**Fig. 4:**
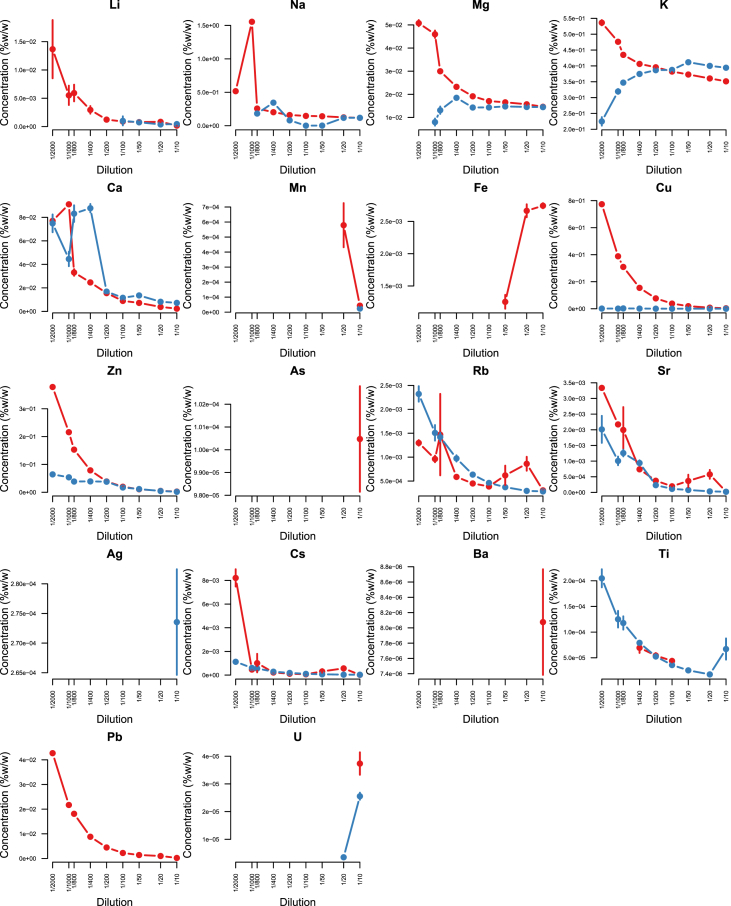
EMSURE® (red line) and SUPRAPUR® (blue line)-digested brain samples.

**Fig. 5:**
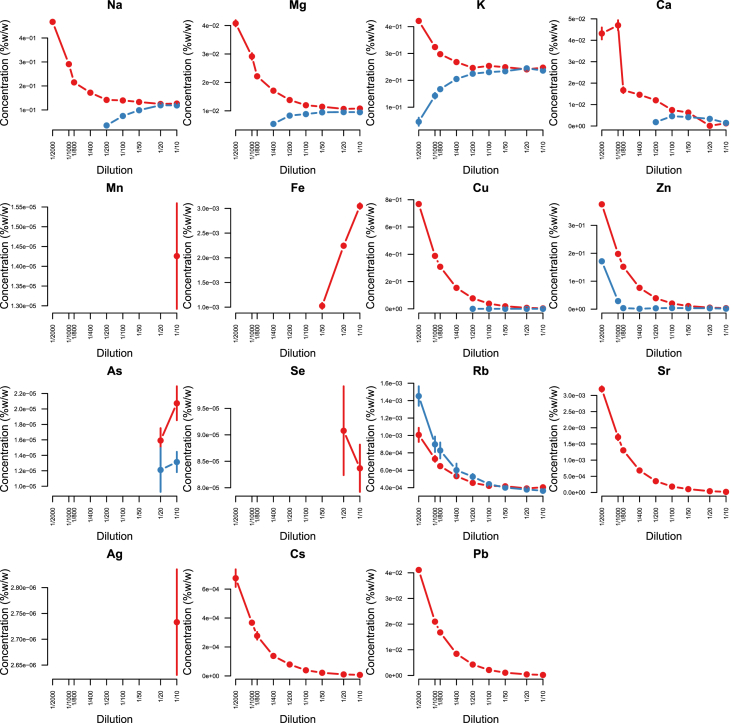
EMSURE® (red line) and SUPRAPUR® (blue line)-digested testis samples.

**Fig. 6:**
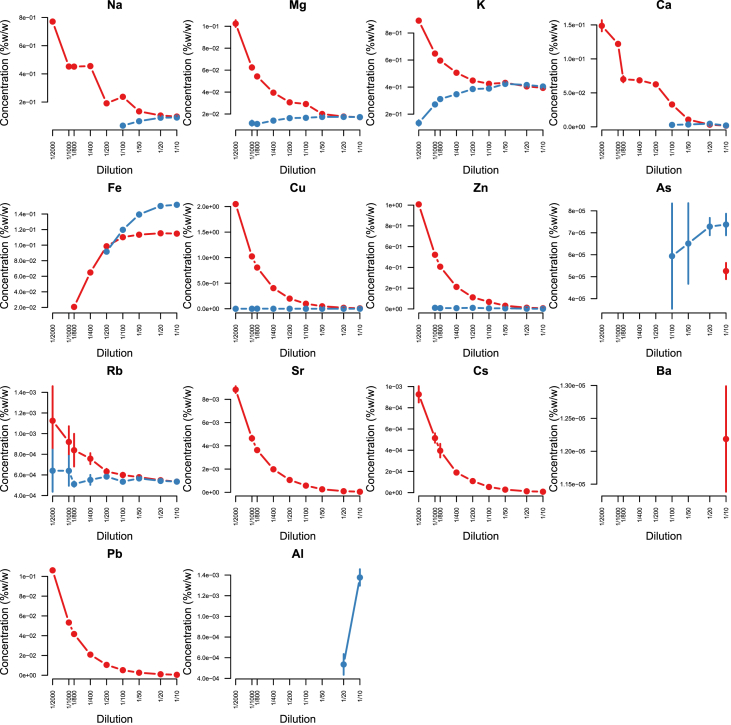
EMSURE® (red line) and SUPRAPUR® (blue line)-digested spleen samples.

**Fig. 7:**
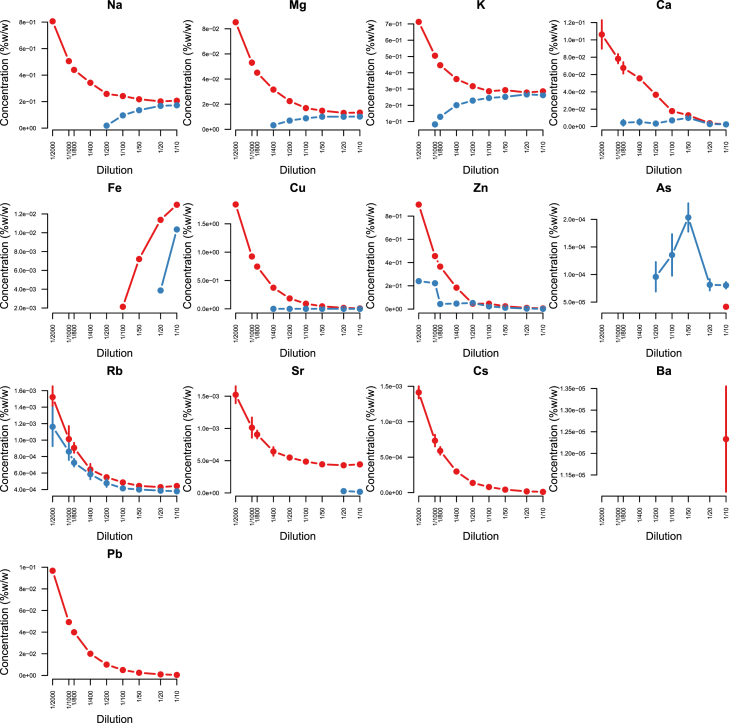
EMSURE® (red line) and SUPRAPUR® (blue line)-digested lung samples.

**Fig. 8:**
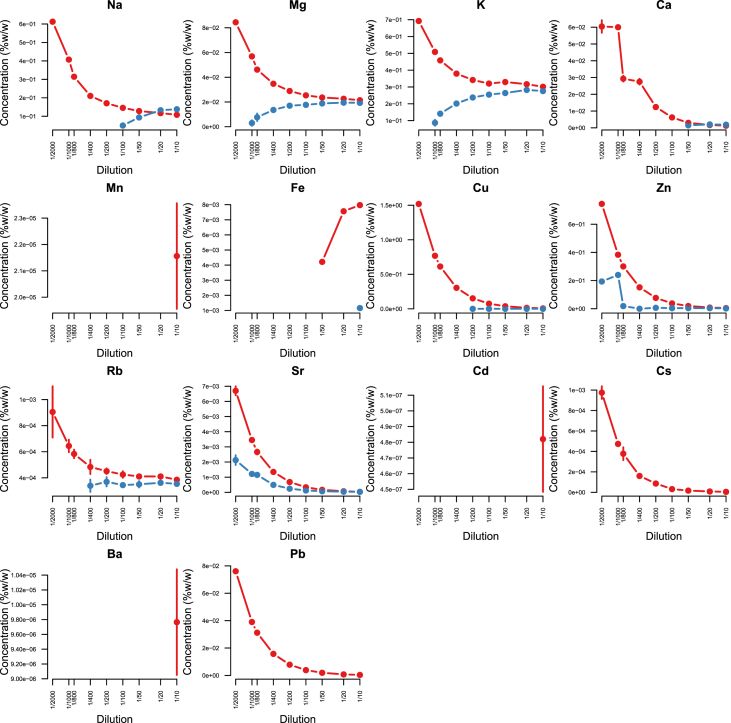
EMSURE® (red line) and SUPRAPUR® (blue line)-digested heart samples.

Minerals and trace elements are essential since they play vital role in the biological functions for all organisms including human and other mammals, since they involve in the structure of various enzymes responsible for detoxification system, amino acid metabolism, immune system and DNA metabolism [[1], [2], [3], [4], [5], [6]]. ICP-MS is the most effective technique to measure trace elements and minerals in the biological samples [7]. In this report we firstly compare efficacy and accuracy of alkali dilution and acidic digestion methods in mammalian samples including brain, liver, testis, lung, kidney, heart, spleen and serum, however alkali digestion was not able to digest tissue samples (supplementary data1). Afterwards we compared acidic digestion with nitric acid (HNO_3_) 65% EMSURE® to SUPRAPUR® grade (Fig. 1, Fig. 2, Fig. 3, Fig. 4, Fig. 5, Fig. 6, Fig. 7, Fig. 8, Table 1, Table 2, Table 3, Table 4, Table 5, Table 6, Table 7, Table 8). EMSURE® grade HNO_3_ resulted in the contamination in the samples (Fig. 1, Fig. 2, Fig. 3, Fig. 4, Fig. 5, Fig. 6, Fig. 7, Fig. 8, Table 1, Table 2, Table 3, Table 4, Table 5, Table 6, Table 7, Table 8). Afterwards we compared different dilution rates to find the optimum one with the lowest RSD value. We found that SUPRAPUR® grade 65% nitric acid is suitable for microwave digestion of serum and tissue samples for ICP-MS analysis. Also, the best dilution rates are 1/10 to 1/20 to obtain the most accurate data according to the relative standard deviation values (Fig. 1, Fig. 2, Fig. 3, Fig. 4, Fig. 5, Fig. 6, Fig. 7, Fig. 8, Table 1, Table 2, Table 3, Table 4, Table 5, Table 6, Table 7, Table 8).

**Table 1:** Mineral and trace element concentrations in rat serum samples.

Dilution rates									
Element (mg/L)	1:10	1:20	1:50	1:100	1:200	1:400	1:800	1:1000	1:2000
EMSURE® Grade									
Na	74 ± 1,2	76 ± 0,8	77 ± 0,7	81 ± 0,5	85 ± 0,7	91 ± 1	93 ± 1,6	97 ± 1,5	121 ± 0,7
Mg	0,064 ± 0,01	0,068 ± 0,05	0,080 ± 0,02	1,1 ± 0,01	1,4 ± 0,02	2,2 ± 0,04	3,4 ± 0,09	4 ± 0,12	7 ± 0,2
K	5,8 ± 0,08	5,9 ± 0,05	6,5 ± 0,04	7,8 ± 0,02	9,8 ± 0,13	13 ± 0,2	22 ± 0,3	26 ± 0,7	45 ± 0,7
Ca	0,5 ± 0,008	0,5 ± 0,01	0,7 ± 0,03	2 ± 0,04	2,7 ± 0,02	6,1 ± 0,2	6,7 ± 0,3	5,1 ± 0,5	7 ± 0,4
Fe	0,1 ± 0,004	0,08 ± 0,002	ND	ND	ND	ND	ND	ND	ND
Cu	0,7 ± 0,006	1,5 ± 0,01	3,8 ± 0,02	7,9 ± 0,06	16 ± 0,12	32 ± 0,28	65 ± 0,24	80 ± 0,94	166 ± 2,1
Zn	0,44 ± 0,004	0,8 ± 0,008	2,4 ± 0,01	4,7 ± 0,03	8,6 ± 0,05	16,6 ± 0,15	32 ± 0,19	42 ± 0,6	81 ± 0,7
Se	0,01 ± 0,001	ND	ND	ND	ND	ND	ND	ND	ND
Rb	0,006 ± 0,0003	0,007 ± 0,0004	0,007 ± 0,0004	0,009 ± 0,0006	0,015 ± 0,001	0,018 ± 0,003	0,03 ± 0,006	0,03 ± 0,006	0,05 ± 0,012
Sr	0,004 ± 0,0002	0,008 ± 0,0002	0,018 ± 0,0002	0,041 ± 0,0009	0,007 ± 0,002	0,15 ± 0,009	0,29 ± 0,007	0,34 ± 0,011	0,67 ± 0,02
Pb	0,041 ± 0,0003	0,084 ± 0,0007	0,2 ± 0,001	0,41 ± 0,002	0,83 ± 0,009	1,69 ± 0,015	3,36 ± 0,001	4,2 ± 0,06	8,8 ± 0,09
SUPRAPUR® Grade									
Na	41,9 ± 0,61	29,6 ± 0,24	28,6 ± 0,28	26,8 ± 0,32	19,5 ± 0,21	10,4 ± 0,24	14,3 ± 0,48	395 ± 2,8	324 ± 2,2
Mg	0,21 ± 0,004	0,14 ± 0,004	28,6 ± 0,28	0,11 ± 0,012	0,069 ± 0,007	ND	ND	ND	ND
K	2,3 ± 0,03	1,5 ± 0,02	1,25 ± 0,04	0,77 ± 0,06	ND	ND	ND	ND	ND
Ca	0,32 ± 0,011	0,22 ± 0,01	0,23 ± 0,02	0,23 ± 0,007	0,01 ± 0,008	ND	ND	ND	ND
Cu	0,016 ± 0,0001	0,011 ± 0,0004	0,008 ± 0,0009	0,005 ± 0,001	0,003 ± 0,003	ND	ND	ND	ND
Zn	0,022 ± 0,001	0,015 ± 0,004	0,13 ± 0,002	0,098 ± 0,01	0,35 ± 0,02	ND	ND	5,1 ± 0,14	11,75 ± 0,18
Rb	0,002 ± 0,0001	0,002 ± 0,0001	0,002 ± 0,0004	0,002 ± 0,0005	0,003 ± 0,001	0,004 ± 0,002	0,005 ± 0,005	0,01 ± 0,004	0,015 ± 0,02
Sr	0,001 ± 0,0001	0,002 ± 0,00007	0,003 ± 0,0004	0,007 ± 0,0009	0,01 ± 0,002	0,02 ± 0,005	0,04 ± 0,005	0,07 ± 0,012	0,14 ± 0,011
Ag	0,015 ± 0,0001	0,008 ± 0,0004	0,004 ± 0,0004	ND	ND	ND	ND	ND	ND

**Table 2:** Mineral and trace element concentrations in rat kidney samples.

Dilution Rates									
Element (mg/L)	1:10	1:20	1:50	1:100	1:200	1:400	1:800	1:1000	1:2000
EMSURE® Grade									
Na	24 ± 0,4	25,5 ± 0,3	33,1 ± 0,2	32,2 ± 0,28	48,8 ± 0,44	37,4 ± 0,21	46,8 ± 0,71	98,1 ± 1,19	83,8 ± 0,72
Mg	2,5 ± 0,03	2,6 ± 0,026	3,4 ± 0,26	3,1 ± 0,026	3,9 ± 0,029	4,3 ± 0,064	5,2 ± 0,15	9,4 ± 0,14	9,3 ± 0,38
K	40 ± 0,43	42,2 ± 0,41	44,4 ± 0,58	46 ± 0,32	46,1 ± 0,36	49,8 ± 0,22	56,7 ± 0,86	66,9 ± 1,19	83,8 ± 1,6
Ca	0,23 ± 0,008	32 ± 0,007	2,9 ± 0,068	1,5 ± 0,03	4 ± 0,089	2,9 ± 0,26	3,5 ± 0,4	17,7 ± 0,81	7,86 ± 1,11
Mn	0,01 ± 0,0003	0,008 ± 0,0003	0,03 ± 0,001	ND	ND	ND	ND	ND	ND
Fe	0,91 ± 0,017	0,87 ± 0,012	0,53 ± 0,012	ND	ND	ND	ND	ND	ND
Co	0,002 ± 0,00007	0,0018 ± 0,0007	ND	ND	ND	ND	ND	ND	ND
Cu	0,79 ± 0,009	1,62 ± 0,013	4,25 ± 0,034	8,31 ± 0,065	16,6 ± 0,1	33,2 ± 0,12	65,9 ± 0,44	83,3 ± 0,52	163,1 ± 2,18
Zn	0,6 ± 0,009	1 ± 0,01	3,5 ± 0,003	4,6 ± 0,005	9,6 ± 0,07	16,9 ± 0,25	32,7 ± 0,34	46,6 ± 0,6	81,5 ± 0,85
Rb	0,07 ± 0,001	0,07 ± 0,001	0,07 ± 0,0009	0,07 ± 0,001	0,07 ± 0,002	0,08 ± 0,003	0,08 ± 0,006	0,09 ± 0,01	0,12 ± 0,008
Sr	0,041 ± 0,0003	0,084 ± 0,0007	0,2 ± 0,001	0,41 ± 0,002	0,83 ± 0,009	1,69 ± 0,015	3,36 ± 0,001	4,2 ± 0,06	8,8 ± 0,09
Cs	0,0006 ± 6x10^−4^	0,0009 ± 6x10^−4^	0,002 ± 0,0001	0,004 ± 0,0006	0,008 ± 0,003	0,01 ± 0,0008	0,03 ± 0,001	0,04 ± 0,004	0,08 ± 0,01
Pb	0,03 ± 0,0007	0,08 ± 0,0007	0,21 ± 0,002	0,42 ± 0,004	0,84 ± 0,008	1,68 ± 0,01	3,31 ± 0,06	4,17 ± 0,05	8,19 ± 0,03
SUPRAPUR® Grade									
Na	6,8 ± 0,11	6,5 ± 0,07	4,2 ± 0,12	1,8 ± 0,07	ND	ND	ND	ND	ND
Mg	1 ± 0,009	1 ± 0,008	1 ± 0,02	0,96 ± 0,01	0,91 ± 0,04	ND	ND	ND	ND
K	17,5 ± 0,16	17,8 ± 0,17	16,5 ± 0,26	16,1 ± 0,17	15,3 ± 0,12	13 ± 0,35	9,3 ± 0,75	7,1 ± 0,59	ND
Ca	0,11 ± 0,004	0,11 ± 0,01	0,11 ± 0,01	0,12 ± 0,014	0,34 ± 0,042	ND	ND	ND	ND
Mn	0,002 ± 0,0009	0,009 ± 0,0001	ND	ND	ND	ND	ND	ND	ND
Fe	0,46 ± 0,007	0,11 ± 0,007	ND	ND	ND	ND	ND	ND	ND
Cu	0,07 ± 0,001	0,07 ± 0,00009	0,07 ± 0,002	0,06 ± 0,003	0,07 ± 0,004	0,41 ± 0,009	0,35 ± 0,02	ND	ND
Zn	0,19 ± 0,002	0,25 ± 0,002	0,52 ± 0,011	0,099 ± 0,011	1,78 ± 0,05	1,09 ± 0,035	1,09 ± 0,048	5,3 ± 0,14	11,6 ± 0,29
Rb	0,029 ± 0,0004	0,029 ± 0,0004	0,029 ± 0,0001	0,028 ± 0,001	0,030 ± 0,002	0,028 ± 8x10^−4^	0,024 ± 0,002	ND	ND

**Table 3:** Mineral and trace element concentrations in rat liver samples.

Dilution Rates									
Element (mg/L)	1:10	1:20	1:50	1:100	1:200	1:400	1:800	1:1000	1:2000
EMSURE® Grade									
Na	5,3 ± 0,07	5,5 ± 0,1	7,1 ± 0,09	7,6 ± 0,01	16,8 ± 0,12	20,3 ± 0,21	30,7 ± 0,51	32,6 ± 1,24	61,8 ± 4,3
Mg	1,24 ± 0,02	1,3 ± 0,01	1,4 ± 0,01	1,6 ± 0,01	2,1 ± 0,04	2,9 ± 0,08	4,1 ± 0,08	4,5 ± 0,2	7,9 ± 0,2
K	21,5 ± 0,9	22,3 ± 0,25	23,4 ± 0,23	22,9 ± 0,13	25,9 ± 0,2	31,6 ± 0,3	38 ± 1,1	40 ± 0,67	59,8 ± 1,5
Ca	0,13 ± 0,006	0,3 ± 0,007	0,64 ± 0,017	0,99 ± 0,04	3 ± 0,13	5,8 ± 0,17	6,9 ± 0,33	4,3 ± 0,45	8,3 ± 0,79
Mn	0,012 ± 0,0001	0,008 ± 0,0003	ND	ND	ND	ND	ND	ND	ND
Fe	0,6 ± 0,009	0,4 ± 0,007	0,12 ± 0,012	ND	ND	ND	ND	ND	ND
Cu	0,73 ± 0,01	1,5 ± 0,02	3,9 ± 0,02	7,9 ± 0,08	16,3 ± 0,09	32,6 ± 0,28	65,5 ± 0,49	81,5 ± 0,6	161,7 ± 1,33
Zn	0,49 ± 0,008	0,87 ± 0,01	2 ± 0,02	4 ± 0,06	8,3 ± 0,06	16,2 ± 0,21	32 ± 0,22	39,5 ± 0,48	79,1 ± 1,22
Rb	0,05 ± 0,001	0,5 ± 0,0005	0,05 ± 0,001	0,05 ± 0,001	0,06 ± 0,001	0,07 ± 0,004	0,08 ± 0,009	0,09 ± 0,009	0,13 ± 0,85
Sr	0,003 ± 0,0002	0,007 ± 0,0002	0,019 ± 0,0004	0,037 ± 0,001	0,07 ± 0,002	0,15 ± 0,002	0,29 ± 0,023	0,034 ±6x10^−4^	0,68 ± 0,034
Cs	0,0008 ± 5x10^−5^	0,001 ± 9x10^−5^	0,003 ± 0,0001	0,006 ± 0,0005	0,012± 7x10^−3^	0,15 ± 0,002	0,05 ± 0,004	0,006 ± 0,002	0,12 ± 0,007
Ba	0,0008 ± 0,00007	ND	ND	ND	ND	ND	ND	ND	ND
Pb	0,039 ± 0,0004	0,08 ± 0,0008	0,2 ± 0,003	0,4 ± 0,005	0,8 ± 0,009	1,7 ± 0,02	3,4 ± 0,02	4,2 ± 0,03	8,3 ± 0,09
U	0,00008±7X10^−6^	ND	ND	ND	ND	ND	ND	ND	ND
SUPRAPUR® Grade									
Na	6,5 ± 0,09	6,1 ± 0,09	4 ± 0,07	1,8 ± 0,08	ND	ND	ND	ND	ND
Mg	1,5 ± 0,01	1,5 ± 0,02	1,5 ± 0,01	1,5 ± 0,04	1,3 ± 0,06	0,8 ± 0,09	0,8 ± 0,1	ND	ND
K	26,2 ± 0,3	26,4 ± 0,2	24,8 ± 0,2	24,6 ± 0,2	11,9 ± 0,19	21,8 ± 0,48	15,6 ± 0,2	14,6 ± 1	3,5 ± 2,1
Ca	0,2 ± 0,007	0,1 ± 0,015	ND	ND	ND	ND	ND	ND	ND
Mn	0,01 ± 0,0001	0,01 ± 0,0005	0,01 ± 0,001	0,004 ± 0,0009	ND	ND	ND	ND	ND
Fe	0,7 ± 0,004	0,8 ± 0,007	ND	ND	ND	ND	ND	ND	ND
Cu	0,03 ± 0,0003	0,03 ± 0,0003	0,03 ± 0,0009	0,03 ± 0,001	0,02 ± 0,002	0,03 ± 0,002	0,02 ± 0,01	0,04 ± 0,02	ND
Rb	0,06 ± 0,0006	0,06 ± 0,001	0,06 ± 0,001	0,06 ± 0,001	0,06 ± 0,001	0,06 ± 0,005	0,06 ± 0,008	0,07 ± 0,008	0,09 ± 0,01
Sr	0,01 ± 0,00009	0,002 ± 0,0002	0,004 ± 0,0003	0,008 ± 0,0009	0,01 ± 0,002	0,02 ± 0,004	0,05 ± 0,005	0,06 ± 0,009	0,12 ± 0,02
Al	0,8 ± 0,01	0,7 ± 0,009	0,49 ± 0,01	0,18 ± 0,01	ND	ND	ND	ND	ND
Cr	0,07 ± 0,0001	0,06 ± 0,0002	ND	ND	ND	ND	ND	ND	ND

**Table 4:** Mineral and trace element concentrations in rat brain samples.

Dilution Rates									
Element (mg/L)	1:10	1:20	1:50	1:100	1:200	1:400	1:800	1:1000	1:2000
EMSURE® Grade									
Li	0,03 ± 0,002	0,1 ± 0,04	0,16 ± 0,04	0,18 ± 0,02	0,25 ± 0,08	0,62 ± 0,14	1,2 ± 0,3	1,2 ± 0,4	2,8 ± 1
Na	24,6 ± 0,32	26,1 ± 0,36	29,6 ± 0,17	30,6 ± 0,36	33,4 ± 2,17	42,3 ± 0,3	53,7 ± 0,74	325 ± 5,25	108 ± 2,9
K	73,4 ± 0,98	75,3 ± 0,55	77,9 ± 0,36	79,8 ± 0,72	82,7 ± 1,1	84,9 ± 0,62	90,9 ± 0,62	99,5 ± 0,38	112 ± 1,7
Ca	0,48 ± 0,012	0,79 ± 0,044	1,5 ± 0,03	1,8 ± 0,07	3,2 ± 0,15	5,15 ± 0,21	6,9 ± 0,58	19 ± 0,2	16 ± 0,93
Mn	0,09 ± 0,0003	0,12 ± 0,031	ND	ND	ND	ND	ND	ND	ND
Fe	0,57 ± 0,008	0,55 ± 0,021	0,26 ± 0,022	ND	ND	ND	ND	ND	ND
Cu	0,7 ± 0,007	1,6 ± 0,03	4 ± 0,03	8 ± 0,1	16 ± 0,13	32,4 ± 0,28	64,7 ± 0,4	81,2 ± 0,73	161,7 ± 1,61
Zn	0,53 ± 0,007	1 ± 0,027	2,2 ± 0,03	4,1 ± 0,06	8,1 ± 0,07	16,5 ± 0,26	32 ± 0,22	45,1 ± 0,59	39,5 ± 0,28
As	0,02 ± 0,0004	0,5 ± 0,0005	ND	ND	ND	ND	ND	ND	ND
Rb	0,06 ± 0,0006	0,18 ± 0,03	0,12 ± 0,04	0,08 ± 0,003	0,09 ± 0,004	0,12 ± 0,01	0,3 ± 0,17	0,2 ± 0,014	0,27 ± 0,01
Sr	0,004 ± 0,0001	0,12 ± 0,029	0,07 ± 0,04	0,04 ± 0,0001	0,07 ± 0,0007	0,15 ± 0,004	0,41 ± 0,15	0,45 ± 0,011	0,69 ± 0,011
Cs	0,004 ± 0,0003	0,11 ± 0,03	0,064 ± 0,04	0,014 ± 0,0003	0,023 ± 0,001	0,045 ± 0,002	0,21 ± 0,16	0,09 ± 0,002	0,17 ± 0,015
Ba	0,0016 ± 0,0001	ND	ND	ND	ND	ND	ND	ND	ND
Ti	ND	ND	ND	0,009 ± 0,0009	0,011 ± 0,001	0,014 ± 0,002	ND	ND	ND
SUPRAPUR® Grade									
Pb	0,04 ± 0,0003	0,21 ± 0,03	0,29 ± 0,04	0,46 ± 0,002	0,93 ± 0,004	1,8 ± 0,01	3,17 ± 0,17	4,5 ± 0,05	8,9 ± 0,06
U	0,007 ± 0,0008	6,1 ± 0,09	4 ± 0,07	1,8 ± 0,08	ND	ND	ND	ND	ND
Li	0,03 ± 0,005	0,03 ± 0,007	0,06 ± 0,01	0,08 ± 0,06	ND	ND	ND	ND	ND
Na	9,9 ± 0,13	9,9 ± 0,09	8,6 ± 0,06	9,8 ± 0,08	6,7 ± 0,12	28,9 ± 0,49	15,3 ± 0,52	ND	ND
Mg	1,21 ± 0,02	1,22 ± 0,004	1,24 ± 0,02	1,2 ± 0,04	1,2 ± 0,04	1,5 ± 0,04	1,1 ± 0,13	0,67 ± 0,12	0,37 ± 0,13
K	33,1 ± 0,37	33,6 ± 0,46	34,5 ± 0,23	32,5 ± 0,19	32,4 ± 0,21	31,4 ± 0,33	29,1 ± 0,52	26,8 ± 0,65	9,44 ± 0,56
Ca	0,61 ± 0,019	0,68 ± 0,03	1,14 ± 0,04	0,97 ± 0,036	1,4 ± 0,11	7,3 ± 0,29	6,98 ± 0,59	3,7 ± 0,51	6,29 ± 0,63
Mn	0,02 ± 0,00009	ND	ND	ND	ND	ND	ND	ND	ND
Cu	0,02 ± 0,00008	0,02 ± 0,0005	0,03 ± 0,002	0,03 ± 0,003	0,05 ± 0,004	0,11 ± 0,01	0,22 ± 0,01	0,09 ± 0,02	0,12 ± 0,02
Zn	0,14 ± 0,001	0,37 ± 0,005	0,97 ± 0,014	1,44 ± 0,016	3,19 ± 0,02	3,28 ± 0,073	3,24 ± 0,035	4,5 ± 0,14	5,4 ± 0,24
Rb	0,02 ± 0,0006	0,02 ± 0,0009	0,03 ± 0,0007	0,03 ± 0,0003	0,05 ± 0,003	0,08 ± 0,0005	0,11 ± 0,007	0,12 ± 0,013	0,19 ± 0,013
Sr	0,001 ± 0,00008	0,002 ± 0,0001	0,006 ± 0,0003	0,009 ± 0,0006	0,01 ± 0,001	0,07 ± 0,007	0,1 ± 0,009	0,08 ± 0,01	0,16 ± 0,036
Ag	0,022 ± 0,0007	ND	ND	ND	ND	ND	ND	ND	ND
Cs	0,002 ± 0,00007	0,002 ± 0,0001	0,005 ± 0,0003	0,008 ± 0,0002	0,01 ± 0,0006	0,02 ± 0,001	0,04 ± 0,005	0,05 ± 0,006	0,09 ± 0,01
Ti	0,005 ± 0,001	0,001 ± 0,00007	0,002 ± 0,0001	0,003 ± 0,0001	0,004 ± 0,0003	0,006±3x10^−4^	0,009 ± 0,001	0,01 ± 0,001	0,01 ± 0,001
U	0,002 ± 0,0001	0,0002 ± 2x10^−5^	ND	ND	ND	ND	ND	ND	ND

**Table 5:** Mineral and trace element concentrations in rat testis samples.

Dilution Rates									
Element (mg/L)	1:10	2:10	1:50	1:100	1:200	1:400	1:800	1:1000	1:2000
EMSURE® Grade									
Na	26,84 ± 0,3	26,4 ± 0,3	28,25 ± 0,2	29,6 ± 0,2	30 ± 0,1	36,4 ± 0,5	45,6 ± 0,53	61,7 ± 1	99 ± 1,45
Mg	2,2 ± 0,03	2,2 ± 0,01	2,4 ± 0,03	2,5 ± 0,04	2,9 ± 0,02	3,6 ± 0,08	4,69 ± 0,1	6,18 ± 0,24	8,6 ± 0,22
K	52,4 ± 0,6	51 ± 0,6	52,8 ± 0,7	53,8 ± 0,3	52,2 ± 0,3	56,8 ± 0,8	63,1 ± 0,6	68,7 ± 0,5	89,3 ± 0,5
Ca	0,2 ± 0,007	0,5 ± 0,018	1,3 ± 0,03	1,5 ± 0,05	2,5 ± 0,13	3 ± 0,13	3,5 ± 0,2	9,9 ± 0,5	9,1 ± 0,5
Mn	0,003 ± 0,0002	ND	ND	ND	ND	ND	ND	ND	ND
Fe	0,6 ± 0,012	0,4 ± 0,009	0,2 ± 0,013	ND	ND	ND	ND	ND	ND
Cu	0,7 ± 0,008	1,5 ± 0,01	4 ± 0,05	8 ± 0,04	16 ± 0,1	32,6 ± 0,3	65,4 ± 0,5	82,3 ± 0,57	162,9 ± 1,16
Zn	0,7 ± 0,01	1,1 ± 0,01	2,3 ± 0,01	4,2 ± 0,06	8,2 ± 0,07	16,1 ± 0,2	32 ± 0,1	41,9 ± 0,6	79,6 ± 0,7
As	0,004 ± 0,0004	0,003 ± 0,0005	ND	ND	ND	ND	ND	ND	ND
Se	0,017 ± 0,0009	0,019 ± 0,0018	ND	ND	ND	ND	ND	ND	ND
Rb	0,08 ± 0,001	0,08 ± 0,001	0,08 ± 0,003	0,08 ± 0,001	0,09 ± 0,003	0,11 ± 0,005	0,13 ± 0,003	0,15 ± 0,01	0,21 ± 0,017
Sr	0,004 ± 0,0001	0,008 ± 0,0002	0,022 ± 0,0007	0,03 ± 0,0008	0,074 ± 0,004	0,145 ± 0,005	0,276 ± 0,008	0,36 ± 0,02	0,67 ± 0,017
Cs	0,001 ± 0,00005	0,002 ± 0,0002	0,004 ± 0,0002	0,008 ± 0,0004	0,016 ± 0,0002	0,029 ± 0,001	0,058 ± 0,005	0,07 ± 0,003	0,14 ± 0,01
Pb	0,04 ± 0,0003	0,08 ± 0,0007	0,22 ± 0,002	0,45 ± 0,005	0,9 ± 0,011	1,79 ± 0,01	3,5 ± 0,004	4,4 ± 0,03	8,7 ± 0,004
SUPRAPUR® Grade									
Na	10,1 ± 0,12	10,2 ± 0,03	8,4 ± 0,06	6,3 ± 0,05	3 ± 0,12	ND	ND	ND	ND
Mg	0,8 ± 0,01	0,8 ± 0,008	0,8 ± 0,015	0,7 ± 0,03	0,7 ± 0,03	0,4 ± 0,05	ND	ND	ND
K	20 ± 0,3	20 ± 0,2	19,8 ± 0,14	19,6 ± 0,12	19 ± 0,2	17 ± 0,14	14 ± 0,39	12 ± 0,9	3,8 ± 1,2
Ca	0,12 ± 0,008	0,28 ± 0,008	0,35 ± 0,04	0,39 ± 0,03	0,15 ± 0,07	ND	ND	ND	ND
Cu	0,01 ± 0,0002	0,013 ± 0,0003	0,013 ± 0,001	0,01 ± 0,001	0,009 ± 0,003	ND	ND	ND	ND
Zn	0,15 ± 0,0009	0,26 ± 0,005	0,29 ± 0,007	0,33 ± 0,007	0,27 ± 0,012	0,12 ± 0,02	0,31 ± 0,05	2,4 ± 0,09	14,5 ± 0,2
As	0,001 ± 0,0001	0,001 ± 0,0002	ND	ND	ND	ND	ND	ND	ND
Rb	0,031 ± 0,001	0,32 ± 0,03	0,034 ± 0,001	0,037 ± 0,0006	0,044 ± 0,002	0,05 ± 0,006	0,07 ± 0,007	0,07 ± 0,007	0,12 ± 0,009

**Table 6:** Mineral and trace element concentrations in rat spleen samples.

Dilution Rates									
Element (mg/L)	1:10	1:20	1:50	1:100	1:200	1:400	1:800	1:1000	1:2000
EMSURE® Grade									
Na	7,7 ± 0,09	8,3 ± 0,12	10,7 ± 0,14	18,9 ± 0,17	15,2 ± 0,13	36,4 ± 0,31	36,1 ± 0,22	36,1 ± 0,73	61,7 ± 0,76
Mg	1,3 ± 0,5	1,4 ± 0,01	1,6 ± 0,028	2,3 ± 0,048	2,4 ± 0,02	3,15 ± 0,08	4,3 ± 0,12	4,9 ± 0,17	8,19 ± 0,24
K	31,5 ± 0,36	32,4 ± 0,35	34,5 ± 0,44	33,9 ± 0,28	35,8 ± 0,28	40,4 ± 0,31	47,7 ± 0,24	51,8 ± 0,35	71,3 ± 0,24
Ca	0,13 ± 0,005	0,25 ± 0,015	0,85 ± 0,029	2,6 ± 0,06	5 ± 0,12	5,48 ± 0,13	5,6 ± 0,36	9,7 ± 0,26	11,8 ± 0,65
Fe	9,1 ± 0,09	9,2 ± 0,12	9 ± 0,08	8,8 ± 0,06	7,8 ± 0,08	5,1 ± 0,12	1,6 ± 0,15	ND	ND
Cu	0,72 ± 0,01	1,5 ± 0,02	3,9 ± 0,03	7,8 ± 0,08	15,9 ± 0,12	32,3 ± 0,28	64,5 ± 0,73	82 ± 0,86	163,9 ± 1,3
Zn	0,54 ± 0,007	0,95 ± 0,013	2,4 ± 0,025	5,4 ± 0,046	8,9 ± 0,096	17 ± 0,17	32,5 ± 0,29	41,7 ± 0,49	80,6 ± 0,87
As	0,004 ± 0,0002	ND	ND	ND	ND	ND	ND	ND	ND
Rb	0,042 ± 0,0007	0,043 ± 0,0005	0,046 ± 0,0008	0,04 ± 0,001	0,05 ± 0,002	0,06 ± 0,004	0,06 ± 0,01	0,07 ± 0,01	0,09 ± 0,02
Sr	0,003 ± 0,0001	0,007 ± 0,0002	0,02 ± 0,0007	0,04 ± 0,001	0,08 ± 0,001	0,15 ± 0,008	0,29 ± 0,01	0,37 ± 0,01	0,7 ± 0,02
Cs	0,0007 ± 0,0002	0,001 ± 0,00006	0,002 ± 0,0002	0,004 ± 0,0004	0,008 ± 0,0009	0,01 ± 0,0008	0,03 ± 0,005	0,04 ± 0,003	0,07 ± 0,006
Ba	0,0009 ± 0,00006	ND	ND	ND	ND	ND	ND	ND	ND
Pb	0,03 ± 0,0002	0,08 ± 0,0005	0,2 ± 0,001	0,41 ± 0,005	0,8 ± 0,009	1,67 ± 0,015	3,3 ± 0,042	4,2 ± 0,05	8,4 ± 0,08
SUPRAPUR® Grade									
Na	6,8 ± 0,12	6,7 ± 0,1	4,8 ± 0,13	2,4 ± 0,06	ND	ND	ND	ND	ND
Mg	1,3 ± 0,01	1,3 ± 0,01	1,3 ± 0,02	1,2 ± 0,03	1,2 ± 0,04	1 ± 0,06	0,8 ± 0,1	0,8 ± 0,1	ND
K	30,7 ± 0,38	31,5 ± 0,36	32,1 ± 0,4	29,6 ± 0,39	29,3 ± 0,21	26,3 ± 0,3	23,6 ± 0,43	20,6 ± 0,97	10,2 ± 1,5
Ca	0,15 ± 0,01	0,34 ± 0,009	0,26 ± 0,007	0,22 ± 0,03	ND	ND	ND	ND	ND
Fe	11,5 ± 0,15	11,4 ± 0,08	10,5 ± 0,23	9 ± 0,04	6,9 ± 0,006	ND	ND	ND	ND
Cu	0,02 ± 0,0003	0,02 ± 0,0005	0,02 ± 0,0006	0,02 ± 0,002	0,03 ± 0,003	0,02 ± 0,008	0,18 ± 0,01	0,02 ± 0,01	0,014 ± 0,02
Zn	0,13 ± 0,001	0,19 ± 0,003	0,45 ± 0,005	0,45 ± 0,01	0,8 ± 0,03	0,6 ± 0,02	0,6 ± 0,06	0,8 ± 0,06	ND
As	0,005 ± 0,0003	0,005 ± 0,0003	0,004 ± 0,001	0,004 ± 0,001	ND	ND	ND	ND	ND
Rb	0,04 ± 0,0005	0,04 ± 0,0008	0,04 ± 0,0008	0,04 ± 0,001	0,04 ± 0,001	0,04 ± 0,003	0,03 ± 0,001	0,04 ± 0,01	0,04 ± 0,01
Al	0,1 ± 0,006	0,04 ± 0,007	ND	ND	ND	ND	ND	ND	ND

**Table 7:** Mineral and trace element concentrations in rat lung samples.

Dilution Rates									
Element (mg/L)	1:10	1:20	1:50	1:100	1:200	1:400	1:800	1:1000	1:2000
EMSURE® Grade									
Na	18,1 ± 0,26	17,7 ± 0,16	19,1 ± 0,19	21,1 ± 0,23	22,7 ± 0,19	30,1 ± 0,39	38,6 ± 0,25	44,5 ± 0,34	70,9 ± 0,7
Mg	1,1 ± 0,02	1,1 ± 0,01	1,3 ± 0,01	1,4 ± 0,02	1,9 ± 0,07	2,7 ± 0,07	3,9 ± 0,09	4,6 ± 0,17	7,5 ± 0,14
K	25,1 ± 0,34	24,5 ± 0,25	25,8 ± 0,16	25,2 ± 0,05	27,9 ± 0,13	31,8 ± 0,15	39,3 ± 0,16	44,4 ± 0,56	62,7 ± 0,89
Ca	0,22 ± 0,004	0,33 ± 0,006	1,14 ± 0,02	1,5 ± 0,04	3,2 ± 0,11	4,8 ± 0,09	5,9 ± 0,5	6,89 ± 0,4	9,3 ± 1,4
Fe	1,1 ± 0,01	1 ± 0,01	0,6 ± 0,02	0,18 ± 0,01	ND	ND	ND	ND	ND
Cu	0,7 ± 0,009	1,5 ± 0,01	3,9 ± 0,03	8 ± 0,05	16,1 ± 0,11	32,8 ± 0,22	65,6 ± 0,37	81,3 ± 0,84	162,1 ± 1,8
Zn	0,52 ± 0,004	0,09 ± 0,01	2,1 ± 0,01	4 ± 0,04	8,1 ± 0,05	16,2 ± 0,12	32,1 ± 0,4	40,2 ± 0,34	79,1 ± 0,98
As	0,003 ± 0,0003	ND	ND	ND	ND	ND	ND	ND	ND
Rb	0,003 ± 0,0008	0,03 ± 0,0004	0,03 ± 0,001	0,04 ± 0,001	0,04 ± 0,001	0,05 ± 0,005	0,08 ± 0,005	0,09 ± 0,01	0,13 ± 0,01
Sr	0,03 ± 0,0008	0,03 ± 0,0004	0,03 ± 0,001	0,04 ± 0,001	0,04 ± 0,001	0,05 ± 0,005	0,08 ± 0,005	0,09 ± 0,01	0,13 ± 0,01
Cs	0,0009± 4x10-5	0,001 ± 0,0001	0,003 ± 0,0001	0,007 ± 0,0002	0,01 ± 0,001	0,02 ± 0,0007	0,05 ± 0,005	0,06 ± 0,01	0,12 ± 0,007
Ba	0,001 ± 0,0001	ND	ND	ND	ND	ND	ND	ND	ND
Pb	0,04 ± 0,0005	0,08 ± 0,0008	0,21 ± 0,0007	0,43 ± 0,004	0,88 ± 0,007	1,7 ± 0,012	3,5 ± 0,04	4,3 ± 0,06	8,5 ± 0,1
SUPRAPUR® Grade									
Na	9,8 ± 0,09	9,5 ± 0,07	7,7 ± 0,11	5,4 ± 0,08	1 ± 0,05	ND	ND	ND	ND
Mg	0,58 ± 0,004	0,57 ± 0,008	0,5 ± 0,007	0,5 ± 0,01	0,39 ± 0,04	0,18 ± 0,025	ND	ND	ND
K	14,9 ± 0,22	15,2 ± 0,11	14,3 ± 0,07	13,9 ± 0,2	13 ± 0,15	11,4 ± 0,23	7,3 ± 0,5	4,7 ± 0,5	ND
Ca	0,14 ± 0,008	0,15 ± 0,019	0,56 ± 0,03	0,4 ± 0,08	0,19 ± 0,07	0,3 ± 0,1	0,3 ± 0,13	ND	ND
Fe	0,59 ± 0,007	0,22 ± 0,01	ND	ND	ND	ND	ND	ND	ND
Cu	0,014 ± 0,0003	0,01 ± 0,0005	0,018 ± 0,0007	0,039 ± 0,002	0,03 ± 0,002	0,03 ± 0,008	ND	ND	ND
Zn	0,11 ± 0,002	0,23 ± 0,004	0,66 ± 0,009	1,3 ± 0,02	2,99 ± 0,04	2,6 ± 0,05	2,5 ± 0,15	12,6 ± 0,2	ND
As	0,004 ± 0,0003	0,004 ± 0,0005	0,01 ± 0,001	0,007 ± 0,002	0,005 ± 0,001	ND	ND	ND	ND
Rb	0,02 ± 0,0004	0,02 ± 0,0001	0,02 ± 0,001	0,02 ± 0,0007	0,02 ± 0,002	0,03 ± 0,003	0,04 ± 0,002	0,04 ± 0,005	0,06 ± 0,01
Sr	0,001 ± 0,00009	0,001 ± 0,0001	ND	ND	ND	ND	ND	ND	ND

**Table 8:** Mineral and trace element concentrations in rat heart samples.

Dilution Rates									
Element (mg/L)	1:10	1:20	1:50	1:100	1:200	1:400	1:800	1:1000	1:2000
EMSURE® Grade									
Na	11,5 ± 0,17	12,5 ± 0,15	13,7 ± 0,13	15,5 ± 0,82	18,2 ± 0,17	22,4 ± 0,18	33,6 ± 0,77	43,5 ± 0,61	65,5 ± 0,84
Mg	2,29 ± 0,03	2,4 ± 0,02	2,5 ± 0,03	2,7 ± 0,04	3 ± 0,06	3,7 ± 0,11	4,9 ± 0,15	6 ± 0,16	9 ± 0,17
K	32,2 ± 0,56	33,8 ± 0,39	35,2 ± 0,22	34,2 ± 0,31	36,5 ± 0,37	40,6 ± 0,27	49 ± 0,42	54,4 ± 0,42	74,1 ± 0,48
Ca	0,13 ± 0,007	0,17 ± 0,009	0,31 ± 0,026	0,66 ± 0,029	1,32 ± 0,11	2,9 ± 0,17	3,1 ± 0,18	6,4 ± 0,08	6,4 ± 0,4
Mn	0,002 ± 0,0002	ND	ND	ND	ND	ND	ND	ND	ND
Fe	0,8 ± 0,01	0,8 ± 0,01	0,45 ± 0,01	ND	ND	ND	ND	ND	ND
Cu	0,77 ± 0,012	1,5 ± 0,01	4 ± 0,04	8 ± 0,1	16,3 ± 0,12	32,6 ± 0,3	65,6 ± 0,7	82,2 ± 0,77	162,5 ± 1
Zn	0,5 ± 0,006	0,89 ± 0,008	2 ± 0,022	4 ± 0,05	8,2 ± 0,06	16 ± 0,08	32 ± 0,44	41 ± 0,24	79,7 ± 0,44
Rb	0,04 ± 0,0003	0,04 ± 0,0007	0,04 ± 0,001	0,04 ± 0,002	0,04 ± 0,002	0,05 ± 0,005	0,06 ± 0,003	0,06 ± 0,005	0,09 ± 0,02
Sr	0,003 ± 0,0001	0,007 ± 0,0002	0,01 ± 0,0002	0,03 ± 0,001	0,07 ± 0,001	0,14 ± 0,005	0,28 ± 0,01	0,36 ± 0,008	0,71 ± 0,03
Cd	0,00005± 3x10-6	ND	ND	ND	ND	ND	ND	ND	ND
Cs	0,0005 ± 5x10-5	0,0008 ± 8x10-5	0,001 ± 0,0001	0,003 ± 0,0003	0,009 ± 0,0002	0,01 ± 0,0009	0,04 ± 0,006	0,05 ± 0,02	0,1 ± 0,006
Ba	0,001 ± 0,00007	ND	ND	ND	ND	ND	ND	ND	ND
Pb	0,03 ± 0,0003	0,08 ± 0,0008	0,2 ± 0,001	0,41 ± 0,006	0,84 ± 0,008	1,6 ± 0,01	3,3 ± 0,03	4,1 ± 0,002	8,1 ± 0,14
SUPRAPUR® Grade									
Na	7,8 ± 0,06	7,5 ± 0,06	5,3 ± 0,07	2,8 ± 0,08	ND	ND	ND	ND	ND
Mg	1,1 ± 0,01	1,1 ± 0,02	1 ± 0,01	1 ± 0,02	0,9 ± 0,03	0,77 ± 0,09	0,43 ± 0,17	0,17 ± 0,12	ND
Ca	0,1 ± 0,007	0,1 ± 0,01	0,08 ± 0,01	ND	ND	ND	ND	ND	ND
K	15,7 ± 0,18	16,1 ± 0,34	15 ± 0,17	14,5 ± 0,18	13,5 ± 0,23	11,4 ± 0,31	8 ± 0,31	4,9 ± 1	ND
Fe	0,06 ± 0,002	ND	ND	ND	ND		ND	ND	ND
Cu	0,03 ± 0,0005	0,03 ± 0,0005	0,03 ± 0,001	0,02 ± 0,001	0,02 ± 0,003	ND	ND	ND	ND
Zn	0,12 ± 0,001	0,23 ± 0,002	0,25 ± 0,004	0,22 ± 0,009	0,38 ± 0,009	0,001 ± 0,042	1 ± 0,09	13,6 ± 0,14	11 ± 0,35
Rb	0,02 ± 0,0002	0,02 ± 0,0005	0,019 ± 0,001	0,019 ± 0,0007	0,019 ± 0,002	ND	ND	ND	ND
Sr	0,001 ± 0,00008	0,002 ± 0,0001	0,004 ± 0,005	0,007 ± 0,001	0,01 ± 0,002	0,02 ± 0,003	0,06 ± 0,006	0,06 ± 0,007	0,12 ± 0,018

## 2 Experimental design, materials and methods

### 2.1 Materials

65% nitric acid EMSURE® and SUPRAPUR® grades were supplied from MERCK (Germany). SUPRAPUR® grade is high-purity acid which is suitable for trace element analysis to avoid possible contaminations. The multi-element solution 2A of SPEX CertiPrep was used for the external calibration standard. And the internal standard, which was provided by Agilent, was used for accurate correction due to the non-spectral interferences. It contains 100 ppm 6Li, Sc, Y, In, Tb, and Bi in 5% HNO_3_. Trace element tubes were obtained from BD (USA).

### 2.2 Animal laboratory

24–28 weeks old Wistar albino rats (*Rattus norvecigus*), weighing 350–450 g were purchased from the experimental animals production center Koc University in Istanbul, Turkey. All rats were housed as described by Aydemir et al. [8,9]

### 2.3 Preparation of serum samples

Blood samples were drained into additive-free vacutainers for trace element analysis (BD vacutainer) allowed to clot and centrifuged at 400 g for 20 min at 4 °C. Serums were separated from blood samples and stored at −80 °C until analysis.

### 2.4 Microwave digestion

Tissue and serum samples were dissolved with the acidic treatment in the microwave digestion system (Milestone START D). 30–80 mg of tissue samples dissolved in 10 ml of 65% HNO_3_ of EMSURE® and SUPRAPUR® grades. 100–200 μl of the serum samples was used for digestion. The microwave digestion process occurs in three steps. Firstly, the temperature increases to 150 °C in 15 min, and then secondly, the target temperature is constant for 30 min. The method of microwave digestion was created after several trials. When a higher temperature was applied (more than 200 °C) for the acidic digestion of tissue and serum samples, the machine stopped to work. A medium range of temperature and power (500W) is sufficient to digest biological samples. After taking the samples from the machine, they were stored at −20 °C until ICP-MS measurement.

### 2.5 Preparation of standards

The multi-element solution 2A of SPEX CertiPrep was used as the external calibration standard. The matrix of the solution is 5% HNO_3_. The standard is prepared from high purity single element concentrates of individual elements using Class A laboratory ware to give precise concentrations. It contains 10 ppm of Ag, Al, As, Ba, Be, Ca, Cd, Co, Cr, Cs, Cu, Fe, Ga, K, Li, Mg, Mn, Na, Ni, Pb, Rb, Se, Sr, Tl, U, V, and Zn. 2 ml of 2% HNO_3_ was used as the blank solution for drawing the abacus origin of the calibration curve.

### 2.6 Measurement of trace element and mineral levels via ICP-MS

After microwave digestion, tissue and serum samples were diluted in the ultrapure water (ddH_2_O) by serial dilution starting from 1/10 and diluted to 1/20, 1/50, 1/100, 1/200, 1/400, 1/800, 1/1000 and 1/2000. Trace and mineral elements in rat tissue and serum samples were measured by Agilent 7700x ICP-MS (Agilent Technologies Inc., Tokyo, Japan). Helium gas is used in the collision/reaction cell in order to remove potential polyatomic interferences. MassHunter Work Station software is used for creating the batch as well as the data analysis. FullQuant analysis method was used for the quantification of the data. The analysis mode was chosen as “spectrum” and for each sample, the peak pattern was drawn from 3 points and the measurements were replicated five times.

### 2.7 Measurement of trace element and mineral levels via inductively coupled plasma mass spectrometry (ICP-MS) with different isotopes of copper, zinc, and lead

The first measurements of acidic digestion were carried out with EMSURE® grade nitric acid. The data showed inconsistency for the trace elements of copper, zinc and lead in which the concentration did not change even the dilution rate decreased. Firstly, the batch was prepared for reading out of ^63^Cu, ^66^Zn, and ^208^Pb. Previous studies [10,11] showed that because of the biological matrix effect, spectroscopic interferences in ICP-MS can arise from the interaction of the sample matrix components and the plasma gas. Moreover, most biological fluids, which contain large amounts of organic compounds and inorganic salts (Ca, Na, K), can lead to spectral and non-spectral interferences. Collision/reaction cell technology can reduce the polyatomic interferences, especially in monitoring of trace elements in biological samples [12]. Hsiung and his colleagues determined that spectral interferences can be eliminated by a simple approach of selecting isotope with minimal interferences, in this case, ^65^Cu instead of ^63^Cu and ^68^Zn instead of ^66^Zn [10]. Therefore, the batch was re-run by adding ^63^Cu, ^65^Cu, ^64^Zn, ^66^Zn, ^68^Zn, and in addition ^206^Pb, ^207^Pb, ^208^Pb isotopes because lead concentration also showed an inconsistency during the acidic digestion with EMSURE® nitric acid. Any recovery in observed (supplementary data2).

### 2.8 Alkali dilution of the serum and the tissue samples

For the alkali dilution method, serum and tissue samples were diluted 1:25 with an alkali solution consisting of 2% (w/v) 1-butanol, 0.05% (w/v) EDTA, 0.05% (w/v) triton X-100, 1% (w/v) NH_4_OH as described by Lu et al. [13]. The mixture was sonicated and homogenized with IKA ULTRA-TURRAX for 5 min and centrifuged at 1000 rpm for 2 min before ICP-MS analysis. Samples were diluted with the same ratio as the acidic digested samples from 1/10 to 1/2000.

### 2.9 Statistical analysis

Statistical analyses were performed using R statistical software version 3.5.1 (https://www.r-project.org). For each tissue versus each trace element and mineral correlation, we calculated the sample mean and the sample deviation over three replicates of the nitric acid (65% EMSURE® and SUPRAPUR® grades) digested samples at each dilution rate. We reported these data as mean ± standard error (SE) in the corresponding figures and tables.
