# Supplementary material for: Dataset of the analyzing trace elements and minerals via ICP-MS: Method validation for the mammalian tissue and serum samples
Source: Data Brief. 2020 Feb 3;29:105218. doi: 10.1016/j.dib.2020.105218 (PMC7016227; doi:10.1016/j.dib.2020.105218)
Supplement: Multimedia component 2 [file mmc2.pdf]

To: Editors-In-Chief of Data in Brief
Re: DIB-D-19-02655

I was listed as one of the authors in the manuscript titled "Dataset of the Analyzing Trace Elements and Minerals via ICP-MS: Method validation for the mammalian tissue and serum samples". I helped the other authors to interpret their results, but I think that my contribution is not enough to be listed as an author. Could you please remove my name from this submission? Thanks in advance for your help.

Sincerely,

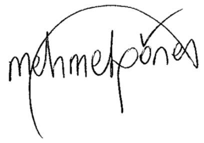

Mehmet Gönen, Ph.D.

Associate Professor of Industrial Engineering, College of Engineering
Associate Professor of Computational Biology, School of Medicine
Koç University, İstanbul, Turkey
mehmetgonen@ku.edu.tr
