# Supplementary material for: Dataset of the analyzing trace elements and minerals via ICP-MS: Method validation for the mammalian tissue and serum samples
Source: Data Brief. 2020 Feb 3;29:105218. doi: 10.1016/j.dib.2020.105218 (PMC7016227; doi:10.1016/j.dib.2020.105218)
Supplement: Multimedia component 1 [file mmc1.pdf]

KOÇ ÜNİVERSİTESİ
SCHOOL OF MEDICINE

Rumelifeneri Yolu
Sarıyer 34450 İstanbul
T: 0212 338 10 00
F: 0212 338 12 05
www.ku.edu.tr

Nuriye Nuray Ulusu, Ph.D.
Full Professor in Biochemistry
Faculty of Medicine, Koc University
Rumelifeneri Yolu, Sarıyer 34450 İstanbul, Turkey

T: +90(212)3381160
F: +90(212)3381168
nulusu@ku.edu.tr
medicine.ku.edu.tr

Dear Editor in Chief Data in Brief,

We have revised our manuscript entitled with "Dataset of the Analyzing Trace Elements and Minerals via ICP-MS: Method validation for the mammalian tissue and serum samples." And manuscript number DIB-D-19-02655.

Mehmet Gonen who is one of the co-author of our manuscript, told us that he has very little contribution to our article. Therefore he remove his name from the authorship and he sent us his request with his signature which you can find it at the attachment.

Authors of the paper, Duygu Aydemir, Gulsu Simsek and Nuriye Nuray Ulusu declare that co-author Mehmet Gonen is removed from the author list.

Kindest regards,

Prof. Dr. N. Nuray Ulusu

DUYGU AYDEMIR

GÜLSU ŞİMŞEK

Prof. Dr. N. NURAY ULUSU
